# Impact of Rotational Atherectomy on Endothelial Integrity and Platelet Activation

**DOI:** 10.3390/ijms262411932

**Published:** 2025-12-11

**Authors:** Wojciech Zimoch, Kamila Florek, Michał Błaszkiewicz, Wiktoria Hanna Buzun, Julia Glińska, Zuzanna Zalewska, Karolina Radek, Monika Kasztura, Ewa Anita Jankowska, Krzysztof Reczuch

**Affiliations:** 1 Institute of Heart Diseases, Faculty of Medicine, Wroclaw Medical University, 50-556 Wroclaw, Poland; 2 Jan Mikulicz-Radecki University Hospital, 50-556 Wroclaw, Poland; 3 Students’ Scientific Group of Invasive Cardiology, Institute of Heart Diseases, Wroclaw Medical University, 50-556 Wrocław, Poland; 4 Department of Food Hygiene and Consumer Health Protection, Faculty of Veterinary Medicine, Wroclaw University of Environmental and Life Sciences, 50-375 Wroclaw, Poland

**Keywords:** rotational atherectomy, E-selectin, ICAM-1, P-selectin, CD40 ligand, PF4, endothelial damage, platelet activation

## Abstract

Rotational atherectomy (RA) is an established technique for modifying heavily calcified and fibrotic coronary artery lesions. Despite its efficacy, the use of a high-speed rotating burr can provoke platelet activation and endothelial injury, thereby increasing thrombotic risk, promoting inflammation, and impairing vascular healing. This study investigated the effects of RA and its procedural characteristics on endothelial function and platelet activation by assessing circulating biomarkers. We prospectively analyzed 34 patients undergoing elective RA at a tertiary center. Blood samples were obtained before and 12–24 h after the procedure. Plasma levels of soluble E-selectin, soluble intercellular adhesion molecule-1 (ICAM-1), platelet factor 4 (PF4), P-selectin, and cluster of differentiation 40 ligand (CD40L) were measured. The study population had a mean age of 71 ± 8.9 years, and 73.8% were male. Cardiovascular comorbidities were prevalent, including diabetes (61.9%), hypertension (92.9%), hypercholesterolemia (42.9%), heart failure (45.2%), atrial fibrillation (21.4%), prior PCI (81%), and prior CABG (11.9%). RA significantly increased levels of P-selectin (55.5 ± 26.1 vs. 68.9 ± 26.5, *p* < 0.001), CD40L (2261.3 ± 2489.9 vs. 3602.0 ± 2428.5, *p* = 0.01), and PF4 (6054.7 ± 5751.8 vs. 10,877.6 ± 4979.7, *p* < 0.001). Moreover, mean burr speed correlated with CD40L elevation, while burr-to-artery ratio correlated with E-selectin increase (all *p* < 0.05). RA induces significant platelet activation and endothelial injury, with biomarker changes suggesting correlation with procedural parameters. These findings highlight the biological impact of RA and may inform strategies to optimize the safety of complex PCI.

## 1. Introduction

Coronary artery disease (CAD) is a progressive cardiovascular condition characterized by endothelial injury, lipid accumulation, plaque calcification, and chronic inflammation. As the disease advances, the coronary arteries narrow, resulting in turbulent blood flow that negatively impacts vascular hemodynamics and platelet and endothelial function [1,2,3]. This disturbed flow contributes to further endothelial dysfunction and platelet activation. Activated platelets and injured endothelium release pro-inflammatory cytokines and chemokines, amplifying vascular injury and promoting atherothrombosis and calcification [4,5].

Severe coronary artery calcification is a major negative prognostic factor in patients undergoing percutaneous coronary intervention (PCI). With an aging population and rising rates of comorbidities, plaque calcification is increasingly encountered in clinical practice. Extensive calcification often hinders optimal stent expansion and may lead to complications such as dissection or stent malposition [6]. Therefore, precise lesion preparation in heavily calcified coronary arteries is essential to achieving favorable PCI outcomes. Among several techniques, such as cutting and scoring balloons, very high-pressure balloons, intracoronary lithotripsy, orbital atherectomy, and excimer laser therapy, rotational atherectomy (RA) remains a widely used and highly effective approach to optimize stent delivery and expansion in composite, calcified lesions [7,8]. However, as RA procedures involve the use of a rapidly rotating burr, this technique is considered aggressive and may result in significant platelet activation and endothelial injury, thereby increasing the risk of periprocedural complications and ultimately contributing to poor outcomes. Consequently, this process can increase thrombotic risk, trigger inflammation, and impair the healing of the treated vessels [9,10]. Moreover, it was shown that RA induces platelet activation more than the standard PCI procedure [11]. It is unknown specifically which procedural factors of the RA procedure (burr speed, size, etc.) impact endothelial and platelet function the most. This raises the question of how levels of endothelial- and platelet-related chemokines, as markers of vascular injury, change during the RA procedure, and whether these changes are associated with procedural factors. To address this question, we aimed to evaluate the impact of RA on vascular injury by measuring circulating biomarkers associated with endothelial damage, such as E-selectin, intercellular adhesion molecule-1 (ICAM-1), and platelet activation, including platelet factor 4 (PF4), P-selectin, and CD40 ligand (CD40L), while tracking their changes throughout the procedure.

## 2. Result and Discussion

### 2.1. Baseline Characteristics

An analysis of the patient population characteristics presented that 73.81% of patients were male, while the average age was 71 ± 8.87 years. The most common risk factors in the study group were hypertension (92.86%), diabetes (61.90%), heart failure (45.24%), and hypercholesterolemia (42.86%). A significantly higher percentage of patients had undergone PCI (80.95%) than CABG (11.90%) in the past (Table 1). During the RA procedure, none of the patients were administered Gp2b3a inhibitors.

Regarding procedural characteristics, the mean contrast volume during the index procedure was 218.21 ± 51.36 mL. Other mean procedural parameters included a total number of burr passages of 5.98 ± 4.18, a total drilling time of 160.71 ± 133.77 s, a burr speed of 145.98 ± 4.98 revolutions per minute (RPM), a total stent length of 27.59 ± 16.18 mm, and a burr-to-artery ratio with a mean value of 0.46 ± 0.08 (Table 2).

Upon discharge, 24% of patients after RA were prescribed triple pharmacotherapy. All of the patients were recommended pharmacotherapy involving ASA and a P2Y12 inhibitor, of which 90.48% were recommended clopidogrel and 9.52% were recommended ticagrelor. Moreover, 80.95% were prescribed ACEIs, 61.90% were prescribed diuretics, 14.29% were prescribed nitrates (p.o.), and 21.43% were prescribed anticoagulants (p.o.), such as acenocumarol (9.52%), dabigatran (2.38%), rivaroxaban (7.14%), and apixaban (2.38%) (Table 3).

### 2.2. Endothelial and Platelet Markers Response to the Procedure

Comparing biomarker levels before and after the RA procedure, statistically significant differences were found in P-selectin, CD40L, and PF-4 levels. RA-associated changes presented as follows: P-selectin before: 47.04 (37.32; 73.97) vs. after: 68.91 ± 26.48, CD40L before: 1712.22 (363.90; 3363.94) vs. after: 3602.02 ± 2428.47, and PF4 before: 4734.45 (1437.75; 8151.30) vs. after: 10,877.55 ± 4979.72 (Table 4).

### 2.3. Correlations Between Procedural Characteristics and Biomarkers Changes

Spearman’s rank correlation for procedural characteristics has shown a positive correlation between the mean burr speed and an increase in CD40L (*r* = 0.34; *p* = 0.03). In addition, the burr-to-artery ratio showed a positive correlation with E-selectin (*r* = 0.3886; *p* = 0.0145). The other procedural characteristics that were analyzed are presented in Table 5.

### 2.4. Discussion

The present study provides new insights into the relationship between platelet activation and the RA procedure. In patients undergoing RA, a significant increase was observed in the levels of biomarkers such as P-selectin, CD40L, and PF-4, indicating enhanced disturbances in platelet activation and inflammatory response. These findings suggest that RA impacts endothelial damage and platelet activation. Moreover, PF-4 released from the α-granules of platelets promotes coagulation, among other things, by neutralizing heparin-like molecules. It also acts as a chemoattractant for neutrophils and monocytes and supports the formation of foam cells, which play a key role in the pathogenesis of atherosclerosis [12]. Secondly, P-selectin, present in platelet granules and Weibel–Palade bodies of endothelial cells, participates in leukocyte recruitment to sites of vascular injury and plays a role in platelet aggregation [13]. Importantly, another significant biomarker, CD40, as a ligand for the CD40 receptor, induces the expression of adhesion molecules (such as VCAM-1, ICAM-1, and E-selectin) and activates the cytokine cascade (including IL-6, TNF-α), thus amplifying the inflammatory response [14,15,16]. The elevation in the levels of these markers—PF-4, P-selectin, and CD40L—may reflect the intensification of thrombo-inflammatory processes, which is particularly relevant in the context of complications after percutaneous coronary interventions (PCI). Especially in regard to stent thrombosis, which is the most frequent in the first days or weeks after PCI due to the platelet activation, endothelial damage, and heightened local inflammatory response [17].

In light of our findings, the observed changes in PF-4, P-selectin, and CD40L levels underscore the potent role of patients’ individual risk assessment based on the endothelial and platelet activation biomarkers potential and targeting them with therapeutic strategies aimed at reducing the risk of thrombosis and recurrent cardiovascular events [18].

Similar patterns to those in the endovascular interventions have been observed in recent studies on inflammatory and thrombotic processes in autoimmune diseases, such as rheumatoid arthritis, where mechanisms of rapid platelet activation and early immune response predominate [19,20,21,22]. The importance of endothelial injury and secondary activation of the coagulation system, as demonstrated in our findings following RA, aligns with the evolution of vascular intervention techniques and certain complications associated with flow disturbances and localized inflammatory responses [23].

In our study, ICAM-1 and E-selectin, in contrast to other platelet-related markers such as P-selectin, PF-4, and CD40L, did not show significant changes following RA. This is likely due to their distinct temporal expression profiles and regulatory mechanisms within the endothelium. E-selectin exhibits a transient, cytokine-dependent induction, reaching its peak within a few hours, whereas ICAM-1 displays a more stable and slower expression profile [24,25]. The mechanical endothelial injury caused by RA may not provide a sufficient stimulus for rapid modulation of these molecules, particularly in the context of limited pro-inflammatory cytokine activation. Cytokines such as IL-1β and TNF-α are critical for upregulating ICAM-1 and E-selectin expression [26]. As a result, platelet biomarkers respond dynamically, whereas endothelial adhesion molecules remain relatively stable, reflecting differences in the kinetics and sensitivity of various components of the circulatory system to acute vascular trauma.

Moreover, our study suggested significant correlations between procedural characteristics and changes in levels of inflammatory and pro-thrombotic biomarkers. A positive correlation was found between the mean rotational speed of the burr and the change in CD40L concentration. This may suggest that higher rotational speeds generate a stronger immune response, potentially due to greater endothelial damage and the release of danger-associated molecular patterns (DAMPs). CD40L, as a marker of immune system activation, may in this context serve as an indicator of procedural aggressiveness [14]. These findings may add another argument into the discussion on optimal burr speed during RA.

**Figure 1:**
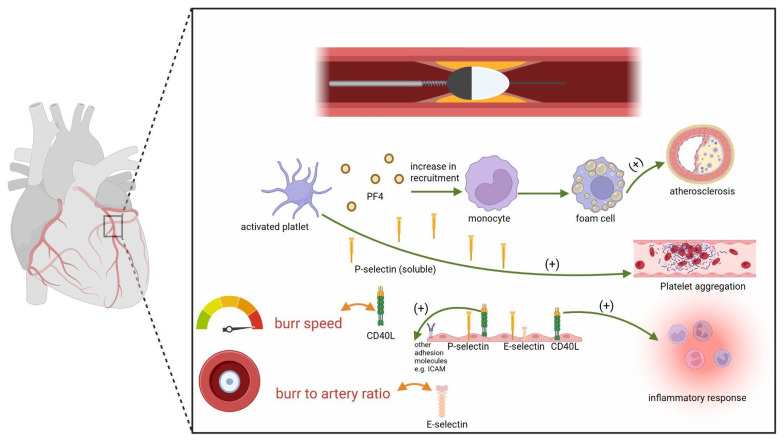
Summary of RA impact on platelet activity and endothelial damage. (+)—process upregulation. Platelet activation leads to the increase in PF-4 and P-selectin levels, which enhances coagulation and supports the formation of foam cells, which play a key role in the pathogenesis of atherosclerosis. CD40L, which level correlated in our study with the burr speed, induces the expression of adhesion molecules—VCAM-1, ICAM-1, and E-selectin—which in particular was associated with burr-to-artery ratio. Moreover, CD40L activates the cytokine cascade, thus enhancing inflammatory response.

Another significant procedural feature, which exhibited a significant correlation, was the burr-to-artery ratio, which positively correlated with the level of E-selectin—an adhesion molecule associated with endothelial activation and damage. A high burr-to-artery ratio is considered a risk factor for microembolization, stronger endothelial activation, and increased inflammation. According to the current expert consensus on RA, a low ratio (0.5–0.6) is recommended to minimize vascular injury [27,28,29]. Hence, our molecular findings align with the currently recommended procedural approach (Figure 1).

Other procedural variables, such as the number of burr passes, total rotablation time, or stent length, did not show significant correlations with the studied markers. This may indicate that it is not the duration of the procedure, but specific technical aspects (such as rotation speed or device size) that are crucial for the biological response of the vessel wall. This study’s small sample size and single-center design may limit the generalizability of the results. Therefore, future multi-center studies with larger cohorts are warranted to validate and extend these findings.

## 3. Materials and Methods

### 3.1. Materials

This was a single-center, prospective observational study that included 34 adult patients hospitalized at a high volume tertiary center. The study protocol was approved by the Bioethics Committee of Wroclaw Medical University and was conducted in accordance with the Declaration of Helsinki (ID no. 827/2020). Informed consent for participation was obtained from all subjects involved in the study.

### 3.2. Clinical Assessment

Upon admission, each patient underwent a comprehensive clinical evaluation, including detailed medical history with assessment of comorbidities, full physical examination, ultrasound imaging, and 12-lead electrocardiography (ECG). Blood samples were also collected for laboratory testing, including WBC, RBC, HGB, PLT, glucose, creatinine, eGFR, and hsTnI.

### 3.3. Procedure

All patients underwent rotational atherectomy (RA) for the modification of calcified atherosclerotic plaques. All of the procedures were performed by the same, experienced team of operators. Procedures were carried out in line with local standards and recommendations, using the RotaLink system (Boston Scientific, Marlborough, MA, USA). Burrs of 1.25 mm, 1.5 mm, and 1.75 mm were used at varying rotational speeds, with detailed procedural decisions left to the operator’s discretion.

### 3.4. Biomarkers

Venous blood samples (10 mL) were collected before the procedure and on the following day (12–24 h post-procedure). All samples were collected at minimum 12 h after administrating standard loading doses of antiplatelets. Samples were stored at −80 °C and secured for further laboratory analysis. Serum concentrations of the following biomarkers were determined: P-selectin, E-selectin, CD40L, intercellular adhesion molecule-1 (ICAM-1), and platelet factor 4 (PF4). Analyses were performed using the enzyme-linked immunosorbent assay (ELISA) with dedicated assay kits for each biomarker (R&D Systems, Minneapolis, MN, USA). Inter-assay coefficients of variation (CV) for every ELISA kit were <12%, whereas intra-assay CV were <10%.

### 3.5. Statistical Analysis

Statistical analyses were performed using Statistica software (version 13.3, Statsoft Polska, Kraków, Poland). The normality of continuous (quantitative) data distribution was assessed using the Shapiro–Wilk test. Normally distributed continuous variables were presented as mean ± standard deviation (SD), while non-normally distributed variables were presented as median with interquartile ranges. Categorical variables were presented using percentages. The statistical significance of changes in biomarkers levels was assessed using the Wilcoxon test, taking into account variations in the normal distribution of data. The relations between changes in biomarker levels and individual continuous or categorical variables were analyzed using Spearman’s rank correlation coefficient. A *p*-value < 0.05 was considered statistically significant.

## 4. Conclusions

RA appears to enhance platelet activity and contribute to endothelial injury. Our exploratory analysis suggested significant correlations between biomarkers’ activity and procedural characteristics, particularly burr speed and the burr-to-artery ratio, which in clinical practice are considered crucial procedural parameters. These results are hypothesis generating; however, further studies in larger cohorts are required to validate these findings and to explore the potential of these biomarkers as prognostic indicators and therapeutic targets.

### Limitations

The presented study has certain limitations. First, it was conducted on a relatively small group of participants, which may limit the generalizability of the findings. To confirm the obtained results and assess their potential in risk stratification and personalized therapy, further research involving multi-center studies with larger and more diverse patient cohorts is required. The study also lacked a comparator group, such as patients undergoing PCI without RA, making the observed association between RA and elevated biomarker levels inferential rather than definitive. Finally, the absence of follow-up data limits the ability to fully assess the prognostic significance of the measured biomarkers.

## Figures and Tables

**Table 1:** Baseline clinical characteristics.

Variable	Values
Age	71 ± 8.87
Gender(M)	73.81%
Diabetes	61.9%
Hypertension	92.86%
Hypercholesterolemia	42.86%
Atrial fibrillation	21.43%
Heart failure	45.24%
Prior PCI	80.95%
Prior CABG	11.9%

**Table 2:** Baseline procedural characteristics.

Variable	Values
Contrast volume, mL, mean (SD)	218.21 (51.36)
Total number of passages, mean (SD)	5.98 (4.18)
Total drilling time, seconds, mean (SD)	160.71 (133.77)
Mean burr speed, RPM, mean (SD)	145,980 (4980)
Total stent length, mm, mean (SD)	27.59 (16.18)
Burr-to-artery ratio, mean (SD)	0.46 (0.08)

**Table 3:** Pharmacotherapy at discharge.

Medication	Values	
ASA	100%	
P2Y12 inhibitor	100%	
	ticagrelor: 9.52%	clopidogrel: 90.48%
Beta-blocker	95.24%	
ACEI	80.95%	
ARB	11.90%	
Diuretic	61.90%	
Statin	97.62%	
Nitrate p.o.	14.29%	
Anticoagulant p.o.	21.43%	
IPP p.o.	66.67%	

**Table 4:** Biomarkers levels changes before and after procedure.

Variable	Before the Procedure	After the Procedure	*p*-Value
P-selectin	47.04 (37.32; 73.97)	68.91 ± 26.48	*p* < 0.001
CD40L	1712.22 (363.90; 3363.94)	3602 ± 2428.71	0.01
PF-4	4734.45 (1437.75; 8151.30)	10,877.55 ± 4979.72	*p* < 0.001
ICAM-1	231.27 (205.41; 286.10)	223.40 (202.16; 296.74)	0.51
E-selectin	27.31 (22.62; 37.66)	29.92 ± 14.71	0.57

Data presentation reflects distribution type: mean ± SD for normal distributions and median (IQR) for skewed distributions.

**Table 5:** Spearman’s rank correlation analysis for procedural characteristics (*r*—correlation coefficient).

	ΔP-Selectin	ΔCD40L	ΔPF-4	ΔICAM-1	ΔE-Selectin
Total number of passages	*r* = 0.0015 *p* = 0.9922	*r* = −0.0976 *p* = 0.5386	*r* = −0.1070 *p* = 0.5001	*r* = −0.0484 *p* = 0.7607	*r* = −0.0323 *p* = 0.8391
Total drilling time	*r* = −0.0573 *p* = 0.7185	*r* = −0.1772 *p* = 0.2617	*r* = −0.1684 *p* = 0.2864	*r* = −0.0006 *p* = 0.9967	*r* = 0.0002 *p* = 0.9992
Mean burr speed	*r* = 0.1214 *p* = 0.4440	***r* = 0.3437** ***p* = 0.0258**	*r* = 0.1676 *p* = 0.2888	*r* = −0.0793 *p* = 0.6179	*r* = 0.0317 *p* = 0.8420
Total stent length	*r* = 0.1362 *p* = 0.3958	*r* = 0.0649 *p* = 0.6868	*r* = 0.1221 *p* = 0.4468	*r* = −0.1469 *p* = 0.3596	*r* = 0.1703 *p* = 0.2872
Burr-to-artery ratio	*r* = 0.1087 *p* = 0.5102	*r* = 0.1327 *p* = 0.4206	*r* = 0.1005 *p* = 0.5426	*r* = 0.2347 *p* = 0.1504	***r* = 0.3886** ***p* = 0.0145**

## Data Availability

The data presented in this study are available on request from the corresponding author.
